# Objective evaluation, using computed tomography, of round window access for cochlear implantation

**DOI:** 10.1007/s00405-024-08873-w

**Published:** 2024-08-04

**Authors:** Katarzyna Radomska, Michał Mielnik, Marcin Gostyński, Edyta Dzięciołowska-Baran

**Affiliations:** 1 Department of Otolaryngology, Pomeranian University of Medicine, Unii Lubelskiej 1, Szczecin, 71-252 Poland; 2 University Clinical Hospital, No. 1, Szczecin, Poland; 3 Department of Anatomy, Pomeranian University of Medicine, Szczecin, Poland

**Keywords:** Artificial intelligence, Cochlear implant, Computer tomography, Robotic surgery, Round window

## Abstract

**Objective:**

The aim of this study was to determine optimal radiological parameters for assessment of the round window approach in cochlear implantation surgery.

**Materials and methods:**

Patients undergoing cochlear implantation at the Department of Otolaryngology in Szczecin, between 2015 and 2022 inclusive, were eligible for the study. Radiological assessments were performed according to eight parameters (seven proposed in the literature) and visibility clinical assessments were made intra-operatively on a scale of 1 to 5 (1 - not visible, 5 - fully visible). Visibility assessments of the round window niche (RWN) and round window membrane (RWM) allowed the difference (RWN minus RWM) to be used as a clinical assessment of the size of the overhang over the round window.

**Results:**

Computed tomography images of 57 ears from 52 patients were analyzed in terms of round window access. The study group included 26 females and 26 males, ranging in age from 1 year to 80 years, with a median age of 41 years. In clinical assessment, round window visibility was rated as 5, after removal of the bone overhang, in 69% of patients. Cochlear access through the round window was achieved in 39 (68%) cases, extended access through the round window in 13 (23%) cases and cochleostomy was performed in 5 (9%) cases. Statistically significant ordinal correlations with round-window access were found using one parameter from the literature (Chen_Angle) and from our proposal (RWM_prediction). From parameters describing the bone overhang of the round window, positive correlations (using Kendall rank tests) were found using parameters from the literature (Sarafraz_OH and Mehanna_OH).

**Conclusions:**

Radiological measurements describing access to the round window which determine the angle based on the anatomy of the posterior wall of the auditory canal and the position of the facial nerve were found to be of the highest value.

**Clinical relevance statement:**

In the future, the use of algorithms for computed tomography evaluation and robot-assisted surgery will require parameters for assessing round window access, for surgery planning and choice of electrode. The parameters proposed by various authors are summarized, allowing researchers to assess their usefulness in further clinical practice.

**Supplementary Information:**

The online version contains supplementary material available at 10.1007/s00405-024-08873-w.

## Introduction

Treatment of hearing loss with cochlear implants is nowadays a common clinical practice. A crucial step in the surgical procedure consists of insertion of the implant electrode through the round window or, alternatively, via cochleostomy. These require precise identification of topographic points, an essential point being the round window niche (RWN) and, after removal of the bone overhang, the round window membrane (RWM), regardless of route of access used.

Cochlear access through the round window (RW) is currently preferred as being less traumatic with a lower risk of electrode translocation to the scala vestibuli, giving better audiological outcomes [1–5] and preservation of vestibular function [6]. Assessment of access to the round window by computed tomography (CT) scanning is crucial in the planning of both classic and robot-assisted cochlear implantation (CI) surgery, as otherwise various complications may appear: such as insertion of the electrode to the hypotympanum, vesibulum or carotid canal [7]. Therefore appropriate measurements, with high intra-observer reliability, can be helpful for both the radiologist and the surgeon.

The development of radiological image evaluation using appropriate and reproducible algorithms requires establishing parameters that will be both relevant and useful in clinical practice. It is also possible that, in the future, cochlear implantation will be performed partially or fully by robots and data concerning radiological evaluation will be also be needed for future algorithm assessment. Given the high anatomical variability within the middle and inner ear, appropriate image positioning prior to the start of measurements is important not only for access evaluation by a clinician but also in cases which might be evaluated by artificial intelligence (AI). If, in the future, imaging of the ear will be performed preliminarily by AI algorithms then these should be equipped with a protocol in which images are aligned according to preset topographic points and in which clinically useful parameters are evaluated. It can be assumed that these AI algorithms will be helpful to radiologists and will speed up the learning curve [8]. The present article has assessed two sets of parameters, one set related to access to the round window (RW) and the second set related to the assessment of the bone overhang over the RW. Of some importance is that few studies have clinically and radiologically assessed the overhang or have separately clinically assessed visibility of the window before and after overhang removal. Currently, the use of an otologic robot mainly consists of using the device for the final stage of surgery i.e. the insertion of an electrode into the cochlea [9, 10]. In parallel, research is ongoing into robotic surgery that will implement other stages of the operation, for example by “keyhole cochlear implantation” [11, 12]. The diameter of the drilling in the latter is slightly larger than the electrode [11], without the procedures of mastoidectomy and posterior tympanotomy. The electrode is inserted through a tunnel drilled previously using a navigation system. For this, precise-access planning is necessary based on imaging studies, especially CT scans [13]. The first procedures in humans using these techniques have been described in the work of Caversaccio et al. [14], on nine cases with three patients requiring intraoperative conversion to classical access via posterior tympanotomy. In the literature, various proposals have been presented for radiological parameters that can be applied in the preoperative evaluation of round window access. Ideally, measurements for the evaluation of round window access should be easy to apply, and be consistent with the clinical assessment made during surgery. The proper alignment of CT images is important and the literature has not always described the necessity of uniformly configuring CT images, correcting for different patient head positions. Some works have described the correction of CT image alignment [15–19], but not all authors have included such corrections in the description of the study methodology [1, 2, 13, 20–29]. In the literature, separate clinical analysis of the visibility of the round window niche, and the round window membrane after removal of the bone overhang, is apparently not available. The surgical step of removing the overhang over the round window involves the use of a drill which potentially exposes the inner ear to acoustic trauma and may contribute to the loss of residual hearing in cochlear implantation procedures used for partial deafness. For this reason, preoperative assessment of the overhang over the round window based on a CT scan is important because it allows planning of the surgery and electrode selection. The aim of this study was to compare the usefulness, in clinical practice, of seven parameters connected with round window evaluation in computed tomography scans for cochlear implantation surgery as proposed by authors of other studies and to analyze an additional parameter proposed by the authors of the present study.

## Materials and methods

The study was completed in accordance with the ethical standards of the Ethics Committee of the Pomeranian University of Medicine in Szczecin (from which approval was obtained) and the principles of the latest World Medical Association Declaration of Helsinki: Ethical Principles for Medical Research involving Human Subjects (2013) including amendments. The trial registration number is KB.006.56.2023. Informed consent was obtained from all subjects and/or their legal guardian(s).

Patients undergoing cochlear implantation at the Department of Otolaryngology in Szczecin, between 2015 and 2022 inclusive, were eligible for the study. Exclusion criteria excluded patients with: obliteration of the round window; inner ear defects; reimplantation; a history of surgery with drilling of the mastoid process on the implanted side; or with signs of otitis media. Inclusion criteria: all patients not excluded; all patients were consecutive patients under the charge of Katarzyna Radomska with agreement for enrollment.

Using high-resolution CT (HRCT) and triplanar reconstructions, CT images were aligned according to a uniform scheme to eliminate differences due to the positioning of the patient’s head during the examination, as follows. The axial plane was acquired in line with the orbito-meatal baseline. The oblique sagittal re-construction was achieved through visualizing the sagittal plane until the view of the lateral semicircular canal was obtained, and this was represented by two dots on its limbs (anterior and posterior); the axial plane was acquired by connecting these dots. The axial data were scrolled till visualization of the summit of the superior semicircular canal (SSC) [15]. All patients, during qualification for cochlear implantation, had CT scans performed in 0.6 mm increments.

**Fig. 1:**
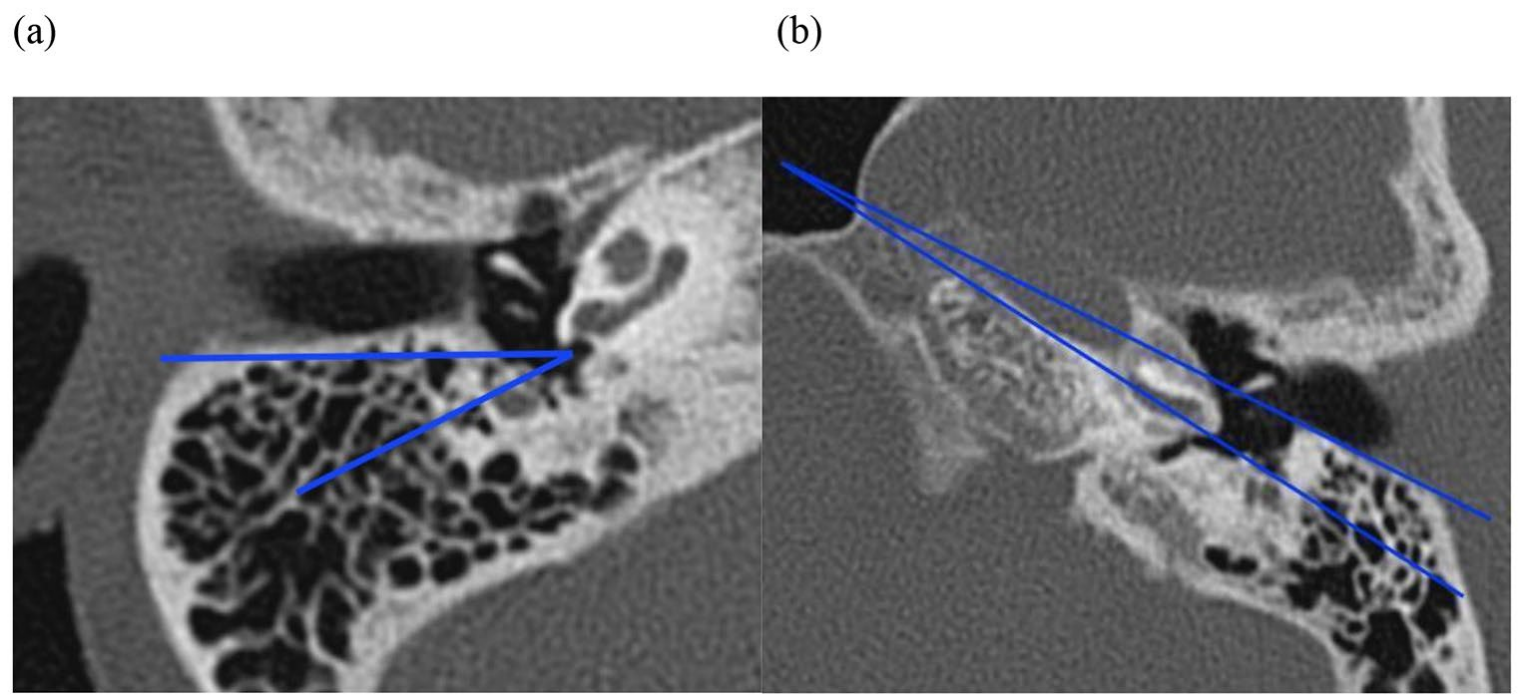
(**a**) Computed tomography scan of the mastoid, right ear. The measurement described by Rajati et al. [18] is indicated by the angle between the two lines. Code: Rajati_Angle. (**b**) Computed tomography of the mastoid, left ear, axial plane. The measurement described by Kashio et al. [20] is indicated by the angle between the two lines. Code: Kashio_Angle

**Fig. 2:**
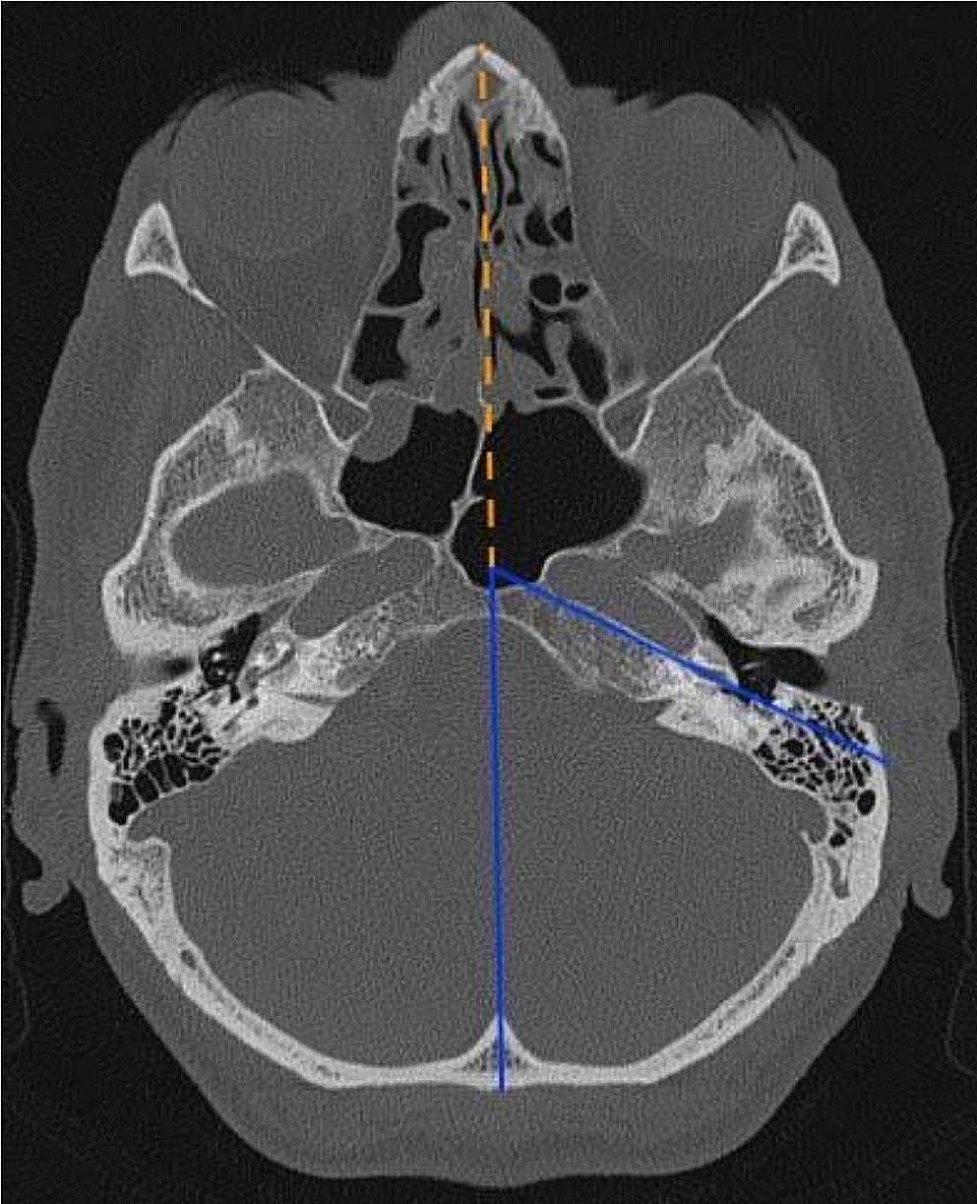
Computed tomography of the ear, both ears can be seen, axial plane. The measurement described by Xie et al. [21] is indicated by the angle between the two lower (solid blue) lines. Code: Xie_Angle

**To evaluate access to the round window, the following radiological measurements were made**:

1. The RWN angle between the line parallel to the coronal axis that passes through the middle of the RWN and the line that connects the anterior portion of the FN to the middle of the RWN, from Rajati et al. [18] (Fig. 1a). Code: Rajati_Angle.
2. The measurement described by Kashio et al. [20] of the angle between a line through the cartilaginous junction in the external auditory canal and the fibrous ring and a second line through the basal turn of the cochlea (Fig. 1b). Code used in this article: Kashio_Angle.

3. Alpha angle from Xie et al. [21]. The angle between the line from the leading edge of the facial nerve (FN) on the plane to the midpoint of the round window membrane and the line from the nasal septum or the perpendicular plate of the ethmoid bone to the occipital protuberance (Fig. 2) [21]. Code: Xie_Angle.

4. The angle proposed by Chen et al. [22] - angular parameter A - between the line drawn from the posterior margin of the RWN and the intersection between mastoid cortex, and the external auditory canal and the line from the posterior margin of the RWN, and the lateral margin of the FN. Code: Chen_Angle.
5. The RWM prediction angle (from authors of the present study). The line drawn along the posterior margin of the external auditory canal and the posterior margin of RWM and line from the posterior margin of the RWN and the anterior portion of the FN. Code: RWM_prediction.

**Fig. 3:**
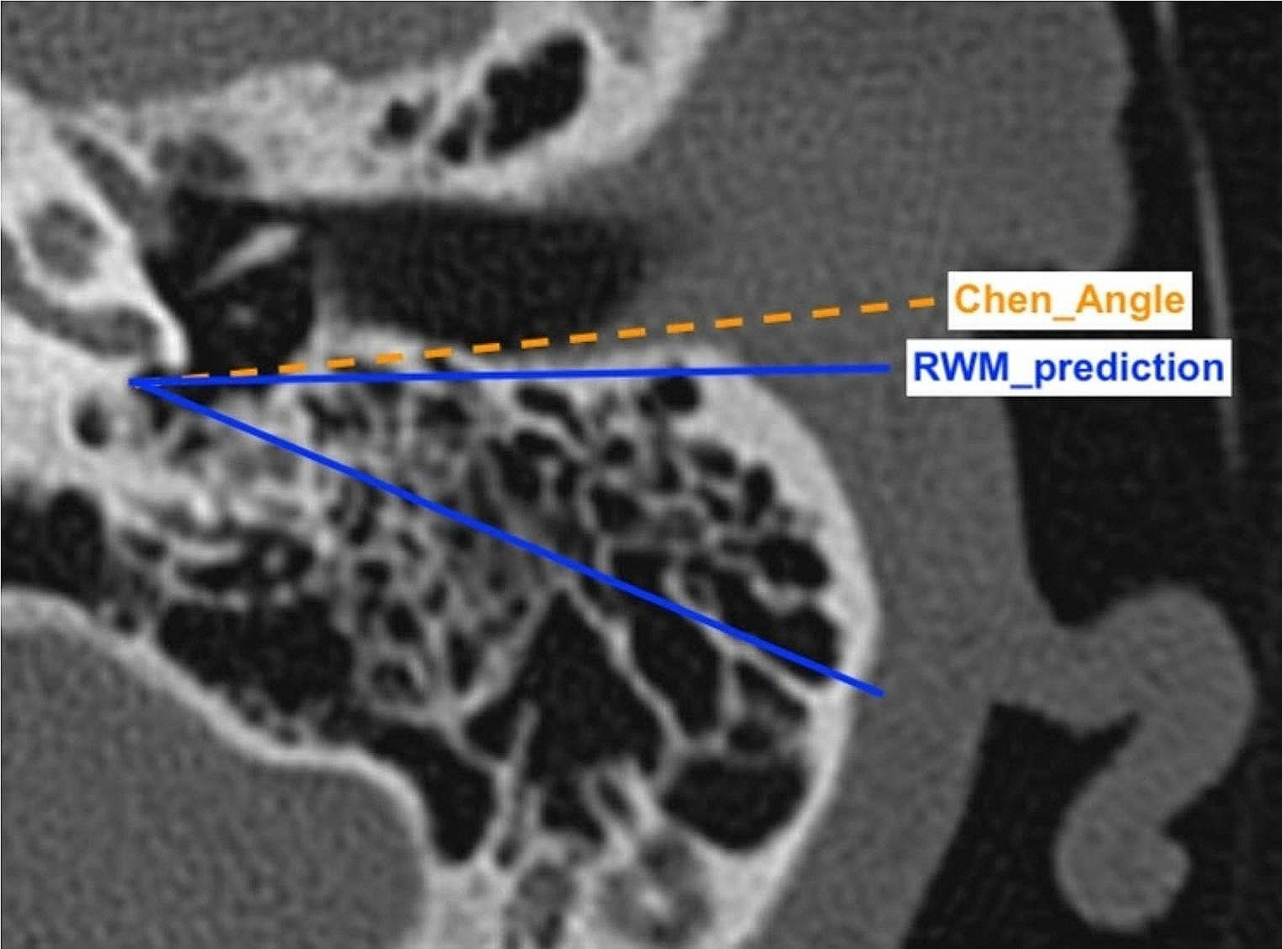
Computed tomography of the mastoid, left ear, axial plane. A comparison of the round window access measurements proposed by Chen et al. [22] - angular parameter A: the angle between the lower (solid blue) line and the (dashed orange) line labelled “Chen_Angle” and the measurement proposed by authors of the present study (RWM_prediction): the angle between the lower (solid blue) line and the (solid blue) line labelled “RWM_prediction”

Based on the above descriptions only, the measurements proposed by Chen et al. (Chen_Angle; [22]) and that proposed by authors of the present study (RWM_prediction) appear similar; to illustrate the difference between these they are shown in Fig. 3.

The second set of assessments consisted of assessing the size of the bone overhang over the round window in order to compare the radiological evaluation with the clinical evaluation:

1. RW bone overhang by Sarafraz et al. [19]. Four consecutive axial (radiological) cuts starting from the highest point of the RW membrane and continuing downwards. The number of cuts in which the bone overhang was identified represented the full thickness of the bone overhang of the round window [19]. Code: Sarafraz_OH.
2. The depth of the RWN, from Mehanna et al. [30]. The distance between the middle of the operculum width and the deepest part of the RWN. Code: Mehanna_OH.
3. The RWN configuration by Mostafa et al. [23] at the level of the cochlear aqueduct: open, hooded or covered. Code: Mostafa_OH.

Evaluations were performed by the senior author (Katarzyna Radomska) and second author (Michał Mielnik). The researchers did not know the results of the clinical evaluation for individual patients during the radiological evaluation.

### Intraoperative assessment of RWN and RWM

**Fig. 4:**
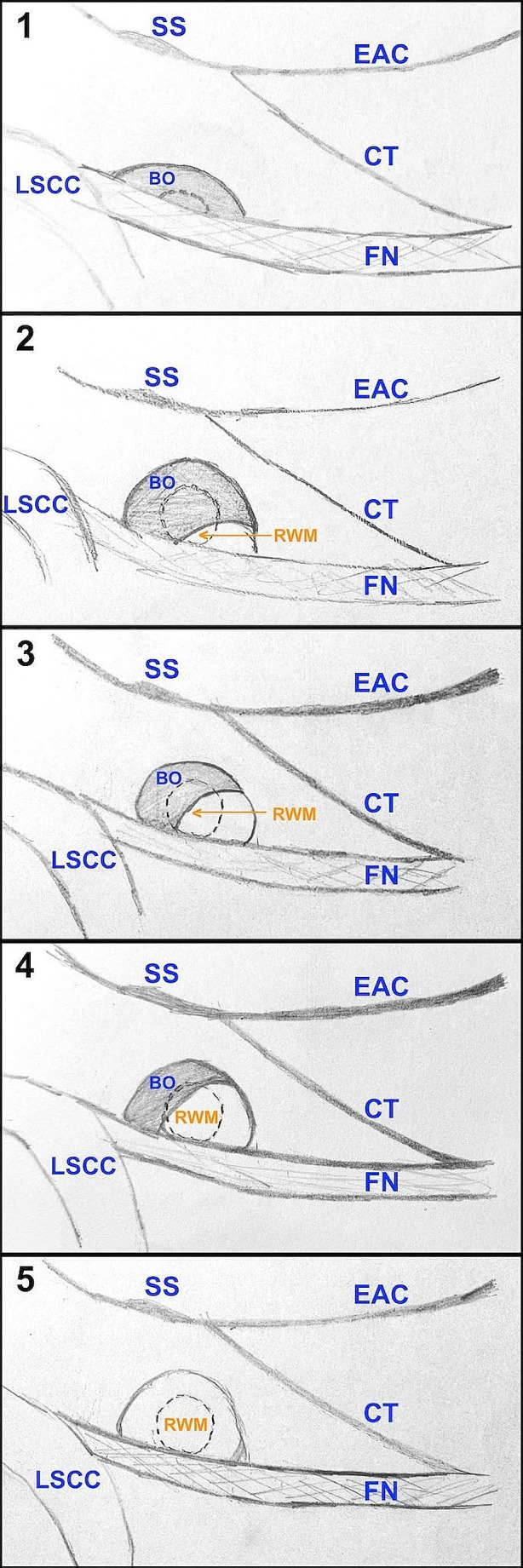
Clinical assessment of RWM visibility. RWM - round window membrane; BO - bone overhang; FN - facial nerve; LSCC - lateral semicircular canal; CT - chorda tympani; EAC - external auditory canal; SS - suprameatal spine

During cochlear implant surgery, intraoperative visibility of the round window niche, after drilling of the bony cells medially to the facial nerve, (code used in this article: InopV_RWN) was assessed in the first stages of surgery and the visibility of the round window membrane (code: InopV_RWM) was assessed after removal of the bone overhang, intraoperatively by the senior author (Katarzyna Radomska). Clinical assessment was made on a 5-grade scale (Fig. 4) where:

5 = visibility of the entire element (RWN or RWM) being assessed;

4 = visibility > 50%;

3 = visibility from 25 to 50%;

2 = upper portion of element visible;

1 = element not visible through posterior tympanotomy.

The difference in the accessibility to the round window niche and the round window membrane (Inop_RWN grade minus InopV_RWM grade) marked the clinical assessment of the size of the overhang over the round window.

### Statistical analyses

All statistical analyses used the R statistical platform [31]. In the statistical analyses two-tailed tests were used and the significance level was set at *p* = 0.05. The p-values were corrected using Benjamini–Yekutieli, i.e. False Discovery Rate (FDR), corrections. These corrections can deal with arbitrary independence (or auto-correlation) between tests (as the sample size was small, the ears were treated as independent cases, rather than individuals). Correlations between the ordinal scales and continuous or ordinal variables were performed using Kendall’s tau correlation coefficients; these non-parametric tests are rank-based and can be used for ordinal data such as the grading scale used in this article [32]. Count plots were created using the R package [ggplot2] function *geom_count* and *scale_size_area* [33]. Associations between the ordinal scales and categorical variables were assessed using Kruskal-Wallis tests and post-hoc Mann-Whitney U tests.

## Results

Computed tomography images of 57 ears from 52 patients were analyzed in terms of round window access. The study group included 26 females and 26 males, ranging in age from 1 year to 80 years, with a mean age of 36 years and a median age of 41 years. In 50/57 (88%) of operated ears, it was necessary to remove the bone overhang medial to the facial nerve, after the posterior tympanotomy had already been performed, for better RW visibility. Results of RW visibility measurements are shown in Table 1.

**Table 1:** Visibility of the round window niche (InopV_RWN) and round window membrane (InopV_RWM) through the posterior tympanotomy, during cochlear implant surgery on 57 ears from 52 patients. The clinical assessment of visibility was graded on a scale of 1 to 5 (see methods; *N* = 57)

Assessment of visibility	InopV_RWN	InopV_RWM
	Number of ears, *n* (%)	
**1**	2 (3%)	0 (0%)
**2**	11 (19%)	3 (5%)
**3**	21 (37%)	3 (5%)
**4**	13 (23%)	12 (21%)
**5**	10 (18%)	39 (69%)

**Fig. 5:**
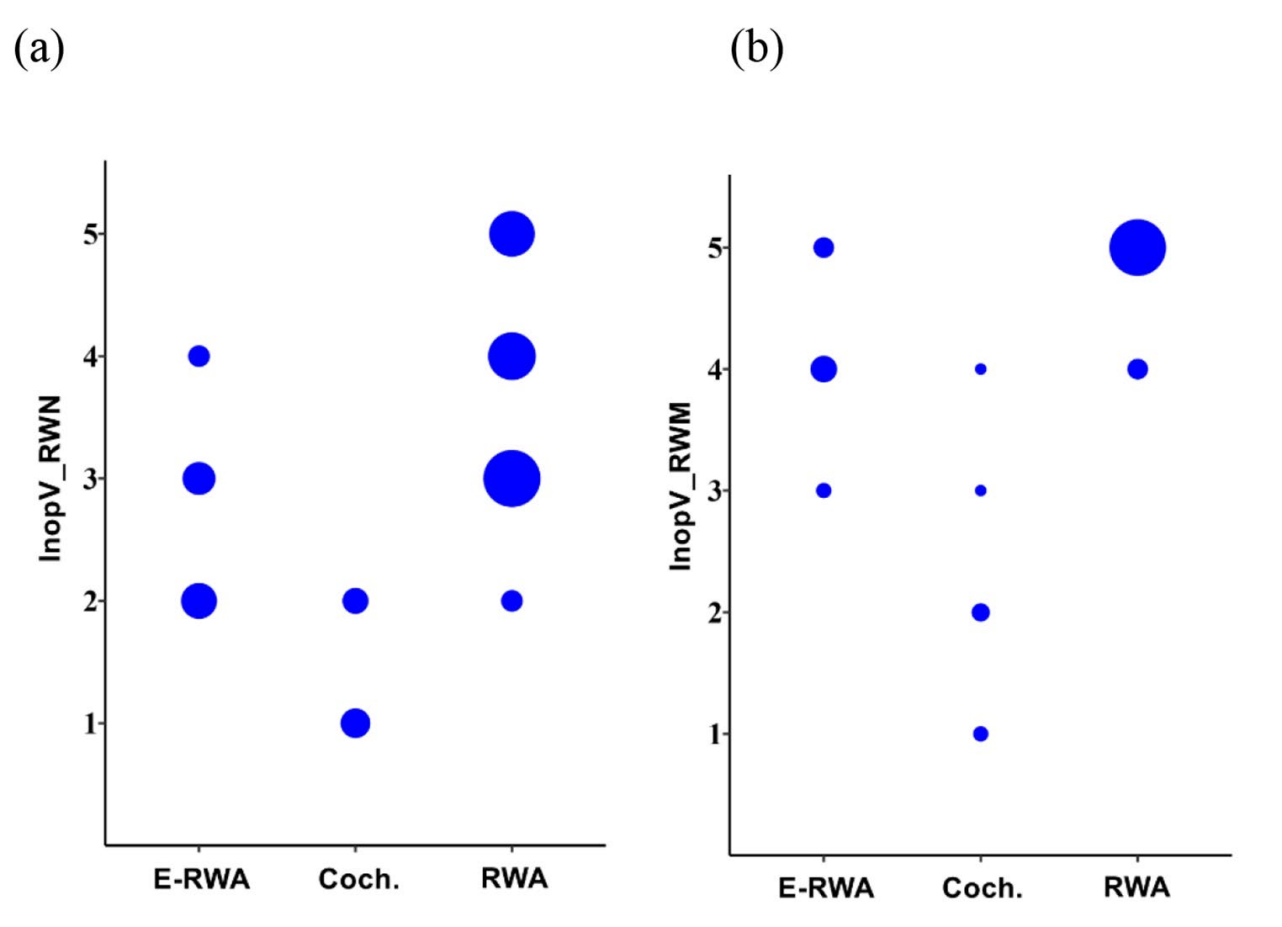
Relationships between the clinical evaluation of round window access and cochlear implant electrode access used. (**a**) Round window niche visibility (InopV_RWN) versus implant access: E-RWA = extended access through the round window; Coch. = cochleostomy; RWA = cochlear access through the round window; (**b**) membrane visibility (InopV_RWM) versus implant access. Count plots are shown: dot area shows relative count. In each graph all three columns show statistically significantly different distributions as assessed by Kruskal-Wallis tests and post-hoc Mann-Whitney U tests: (**a**) Coch. vs. E-RWA *p* = 0.00639, RWA vs. E-RWA *p* = 0.00254, RWA vs. Coch. *p* = 7.08 × 10^− 5^; (**b**) Coch. vs. E-RWA *p* = 0.00387, RWA vs. E-RWA *p* = 5.10 × 10^− 5^, RWA vs. Coch. *p* = 1.10 × 10^− 7^

During the surgical procedure, the bone overhang over the round window was removed and the visibility of the round window membrane was then reassessed. Cochlear access through the round window was performed in 39 patients, extended access through the round window in 13 cases and cochleostomy in 5 patients. Full insertion of the electrode was achieved in all cases. Figure 5 shows the relationship between the clinical evaluation of the RW access and cochlear implant electrode access used.

In all patients whose RW access was graded 3 to 5 (by InopV_RWM or InopV_RWN) the round window or extended round window approach was performed. Cochleostomy was performed in patients whose access (by InopV_RWM and InopV_RWN) was graded as 1 or 2.

In the statistical analyses, after false discovery rate corrections, several Kendall rank correlations were found to be significant; results are shown in Table 2.

**Table 2:** Values of parameters obtained during cochlear implant surgery on 57 ears from 52 patients. For parameter codes see methods. Kendall correlation coefficients are given for associations between five parameters and intraoperative assessment of round window niche (InopV_RWN) and round window membrane (InopV_RWM) access

Parameter code.	Min.	Max.	Mean	S.D.	Median	Kendall rank correlations		
						Coefficient	InopV_RWN	InopV_RWM
Kashio_Angle [⁰]	5.7	38.4	12.6	3.9	9.0	tau	0.184	0.222
						p	0.135	0.099
Xie_Angle [⁰]	40.3	78.7	60.6	8.4	56.6	tau	-0.035	-0.022
						p	0.935	0.874
Rajati_Angle [⁰]	11.1	42.6	26.2	7.9	28.9	tau	0.212	0.206
						p	0.107	0.124
Chen_Angle [⁰]	8.1	40.6	26.8	8.4	27.5	tau	0.260	0.267
						p	0.036	0.042
RWM_prediction [⁰]	6.4	35.3	20.0	9.2	24.8	tau	0.368	0.412
						p	0.001	< 0.001

Min. = minimum; Max. = maximum; S.D. = standard deviation; Kendall’s rank correlation: tau = Kendall’s tau; p = p-value. Kendall parameters (tau and p) to 3 decimal places; other parameters to 1 decimal place; Significant p-values are highlighted

The prediction angle described by Chen et al. [22] and the RWM prediction angle (RWM_prediction) had strong associations with the clinical evaluation of InopV_RWN and InopV_RWM. No statistical relationship was shown for radiological measurements (related to access to the round window) proposed by the other authors. This suggests that as the value of the measured angle increases, a better association with visibility of the round window can be expected.

**Table 3:** Parameter values from radiographic and clinical assessments of the bone overhang size over the round window. Kendall correlation coefficients are given for associations between two parameters and intraoperative assessments of round window niche (InopV_RWN) and membrane (InopV_RWM) access, and InopV_RWN minus InopV_RWM (RWN-RWM)

Parameter code	Min.	Max.	Mean	S.D.	Median	Kendall rank correlations			
						Coefficient	InopV_RWN	InopV_RWM	RWN- RWM
Sarafraz_OH [number of slices]	1	10	3.86	3.12	4.0	tau	-0.495	-0.191	0.548
						p	< 0.001	0.172	< 0.001
Mehanna_OH [mm]	0.27	2.11	1.78	0.64	1.30	tau	-0.348	-0.202	0.316
						p	0.003	0.132	0.011

For abbreviations, see Table 2. Kendall parameters (tau and p) to 3 decimal places; other non-integer parameters to 2 decimal places; Significant p-values are highlighted.

Both of the radiological measurements of the overhang over the RW proposed by Sarafaz et al. (Sarafraz_OH; [19]) and Mehanna et al. (Mehanna_OH; [30]) showed statistically significant correlations with the intraoperative assessments of the niche (InopV_RWN) and the difference between the niche and membrane assessments (InopV_RWN - InopV_RWM). However Sarafraz_OH vs. InopV_RWM was not significant. Results are shown in Table 3. In the present study, the difference in the evaluation before and after removal of the overhang over the window gave the clinical evaluation of the size of the overhang based on intraoperative evaluation.

**Table 4:** Configuration of the round window using a method described by Mostafa et al. [23] (Mostafa_OH)

Parameter	Open	Hooded	Covered
	Number of operated ears, *n* (%)		
Mostafa_OH	17 (30%)	29 (51%)	11 (19%)

**Fig. 6:**
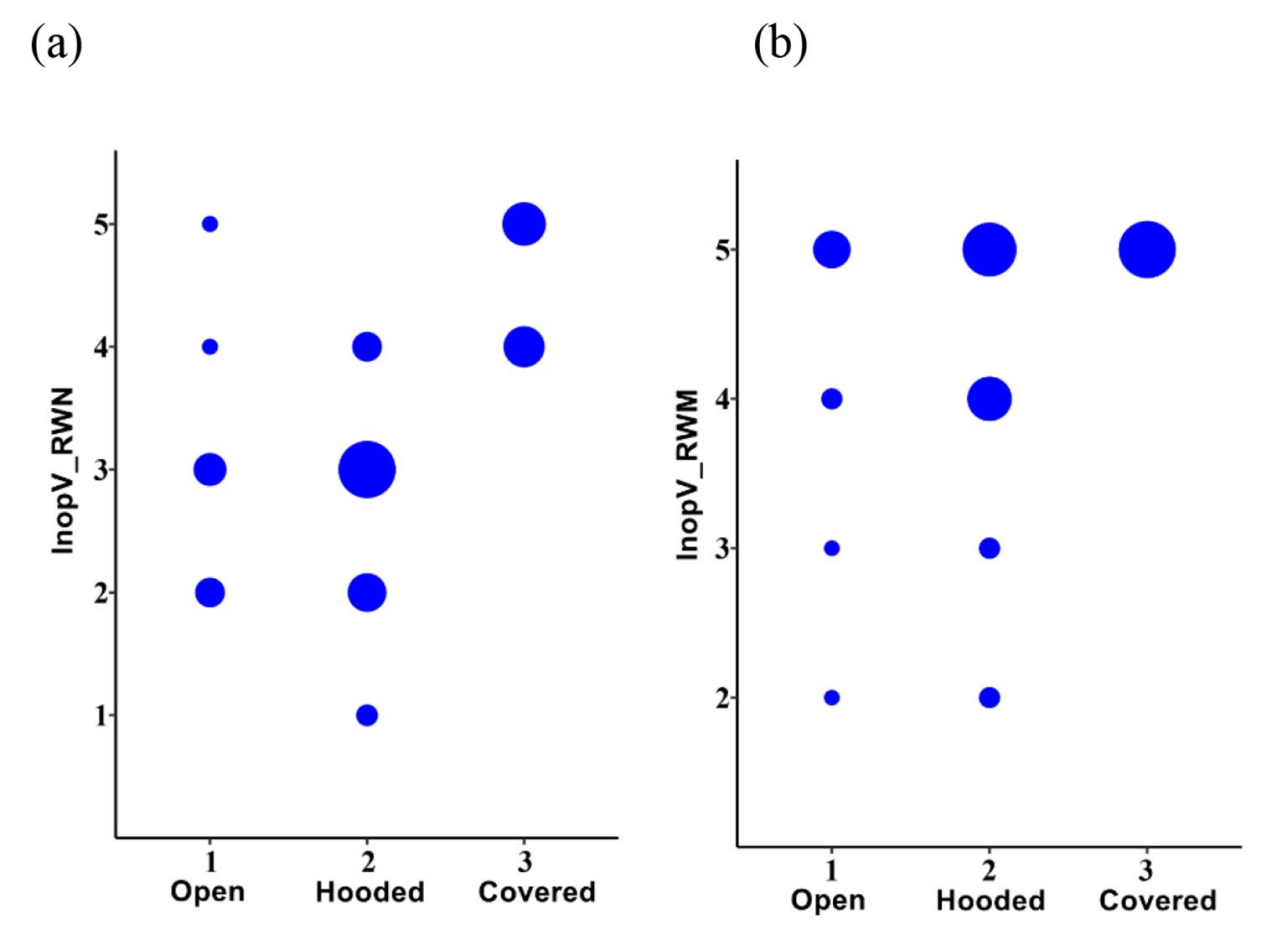
Associations between the round window configuration according to Mostafa (Mostafa_OH) and clinical assessments of (**a**) round window niche (InopV_RWN) and (**b**) membrane (InopV_RWM). Count plots are shown: dot area shows relative count. By treating Mostafa_OH as ordinal with order: open, hooded and then covered, Kendall rank correlations gave statistically significant associations between the variables: (**a**) tau = 0.554, *p* = 1.59 × 10^− 5^; (**b**) tau = 0.317, *p* = 0.0349

Configuration of the RW as according to Mostafa et al. [23] is described in Table 4. From the evaluation of the overhang over the round window there was a noticeable difference in the evaluation of access to the window before and after the overhang had been removed. Even with an initial assessment of poor access to the RW, after the overhang was removed even poorly visible windows could then be visualized. If the window was assessed as covered or hooded using CT, this indicated a greater need for overhang drilling. After removal of the bone overhang, the RWM could be visible even in covered cases. The associations are shown in Fig. 6.

## Discussion

This paper has attempted to evaluate seven parameters proposed to date in the CT radiological evaluation of round window access and the authors present an additional parameter (RWM_prediction) which was developed based on their clinical experience and the other parameters described in the literature.

To facilitate the evaluation of the results, this discussion is divided into sections related to the anatomical structure of the temporal bone.

### Measurements describing access to the round window: angle measurements

Of the parameters which were measured in the present study, an association was only found with round window visibility for the measurements proposed by Chen et al. (Chen_Angle; [22]) and by the authors of the present study (RWM_prediction). In our study, despite measurements by two independent investigators, we could not confirm the usefulness of the measurements by Kashio et al., Xie et al. or Rajati et al. [18, 20, 21].

The measurement described by Kashio et al. (Kasio_Angle; [20]) indicated the possibility of access to the basal turn of the cochlea without evaluation of the round window. The determination of a line through the basal turn is not precise. Our work failed to demonstrate a statistical relationship between the proposed measurement and the evaluation of access to the round window. The measurements proposed by Xie et al. (Xie_Angle; [21]) and Rajati et al. (Rajati_Angle; [18]) are based on the fact that one of the lines runs in a fixed, reproducible way.

In the case of the Xie_Angle, this is the line from the nasal septum or the perpendicular plate of the ethmoid bone to the occipital protuberance. Xie et al. [21] found that as the value of the described angle increased, problems with access to the window appeared to increase. Such a relationship could not be confirmed in our work.

In Rajati et al.‘s measurements [18], one of the lines is determined parallel to the coronal axis. The advantage of such a fixed line is its replicability; however, the anatomical diversity of the temporal bone must be taken into account. It is precisely such diversity in anatomical structure that can be used to explain the fact that our work failed to demonstrate a statistical relationship between the proposed measurements and the clinical assessment of access to both the niche and the round window membrane.

The present study has demonstrated the clinical usefulness of measurements based on the angle contained between the posterior wall of the external auditory canal and the line drawn by the position of the facial nerve and the round window. As the value of the angle between the described lines increased, the visibility of the round window was shown to have improved.

The limitations of the study are:

(1) The small sample size (these were consecutive patients over a period of seven years).

(2) Ears were treated as independent because of the small sample size. With a larger sample size dependence on individual should also be assessed.

(3) This study is from a single-center experience. While this means that all measurements were made by the same team and were therefore more consistent, other teams might have introduced different biases.

In successful associations from measurements as described by Chen et al. [22] (angular parameter A - between lines described by author as lw and lf - which are - lf line - from the posterior margin of the RWM to the lateral margin of the FN - and lw line - the line from the posterior margin of the RWM to the intersection of the posterior wall of the EAC and the mastoid cortex), the cartilaginous-bony junction was designated as the border of the posterior wall of the ear canal, but in radiological measurements it is difficult to determine the precise termination of the cartilaginous part because the description presented by Chen et al. showed the site of soft tissue attenuation which does not always correspond to the termination of the cartilaginous part. The measurement proposed in our work (Code: RWM_prediction) takes into account the anatomy of the posterior wall of the ear canal and more adequately reflects the situations encountered during surgery, which can justify the difference in statistical significance of the two measurements in favor of the angle proposed by the authors of the present study. It should be noted that both the angle measurement proposed by Chen et al. and the measurement described by the present authors meet the criteria for statistical significance and, assuming that subsequent studies replicate our findings, can then be proposed for daily practice. These could also be considered in the future for AI protocols and in the planning of fully robot-assisted cochlear implant surgery. However, given the small sample size of the studied subjects, it seems prudent to recommend further studies with the most clinically-useful measurements and, based on larger groups of patients, to establish ranges of values for each window visibility assessment.

### Parameters determining the overhang over the round window: Mostafa_OH, Sarafraz_OH and Mehanna_OH

Very often there is a need to remove the overhang of the promontory over the round window in order to improve the visibility of the round window membrane [30]. However, neither the presence of a bone overhang nor its size determines the ultimate visibility of the round window membrane or ease of access for the electrode. This was demonstrated in a study by Sarafraz et al. [19] and confirmed by our study. However, for the future of robotic surgery, the mere presence of the overhang over the window may be an issue because it will be necessary to remove this. This could also be important in the planning of surgery as regards hearing preservation, because drilling of the cochlea may lead to acoustic trauma and loss of residual hearing.

In our study, we found a statistically significant correlation between the radiographic evaluation of the overhang over the window and the difference in the clinical evaluation of access to the niche and the round window membrane. In this regard, both the measurements proposed by Sarafraz et al. (Sarafraz_OH) and Mehanna et al. (Mehanna_OH) seem of high value. (Note that the correlation was much stronger for Sarafraz_OH.) In the evaluation of the overhang proposed by Mostafa et al. (Mostafa_OH), it was shown that the description *covered* or *hooded* meant that more overhang removal was required. When milling these fragments, a much slower drill speed was used, but this type of manipulation always meant a greater risk of acoustic trauma to the cochlea and loss of residual hearing.

## Conclusions

In clinical evaluation, radiological measurements describing access to the round window which use determination of the angle based on the anatomy of the posterior wall of the auditory canal and the position of the facial nerve in the mastoid region, are of the highest value. It seems that this approach will be sufficient for insertion of the straight electrodes. However, it will not be sufficient for pre-curved electrodes comprising a stylet or a sheath that has to be inserted into the basal turn. For this type of electrode, the insertion path must be aligned with the long axis of the basal turn. In such cases, the approach of Kashio might be more suitable.

An overhang over the round window is not an obstacle to cochlear implantation, and its size can be predicted on the basis of radiological evaluations determined by the number of radiological scans on which it is present. The size of the study group was insufficient to determine which ranges of radiological parameter values indicate good visibility of the window during surgery. However, it seems reasonable to conduct further multicenter work based on the evaluation of these selected parameters, so that they can be used in future AI algorithms for evaluation of radiological examinations and also for the planning of robot-assisted cochlear implant surgery.

## Electronic supplementary material

Below is the link to the electronic supplementary material.

Supplementary Material 1

## Abbreviations

RW: Round window
CI: Cochlear implant
RWN: Round window niche
RWM: Round window membrane
CT: Computed tomography
AI: Artificial intelligence
HRCT: High-resolution computed tomography
SSC: Superior semicircular canal
FN: Facial nerve
FDR: False Discovery Rate
InopV_RWN: Intraoperative visibility of the round window niche
InopV_RWM: Intraoperative visibility of the round window membrane
Kashio_Angle: Angle measurement described by Kashio et al. [20]
Xie_Angle: Angle measurement described by Xie et al. [21]
Rajati_Angle: Angle measurement described by Rajati et al. [18]
Chen_Angle: Angle measurement described by Chen et al. [22]
RWM_prediction: Angle measurement proposed by the authors of the present study
Sarafraz_OH: Round window bone overhang measurement according to Sarafraz et al. [19]
Mehanna_OH: Round window bone overhang measurement according to Mehanna et al. [30]
Mostafa_OH: Round window bone overhang measurement according to Mostafa et al. [23]
